# Behavioral impact of chemogenetic manipulations of 5-HT DRN neurons in transgenic Tph2-iCre rats

**DOI:** 10.1007/s00213-025-06947-z

**Published:** 2025-11-15

**Authors:** Nicholas S. McCloskey, Chen Li, Lynn G. Kirby

**Affiliations:** Center for Substance Abuse Research, Lewis Katz School of Medicine at Temple University, 3500 N. Broad Street, Philadelphia, PA 19140 USA

**Keywords:** DREADDs, Serotonin, Substance abuse, Dorsal raphe nucleus, Conditioned place preference, Elevated plus maze, Forced swim test, Chemogenetics, Transgenic rat

## Abstract

**Rationale:**

Our laboratory has demonstrated that stressors regulate the 5-HT system with consequences in behavioral models of psychiatric disorders including addiction. We have employed behavioral pharmacology methods to show that stressors engage corticotropin-releasing factor afferents to 5-HT dorsal raphe nucleus (DRN) neurons with effects on negative affective state and stress-induced opioid relapse models. Given the ongoing opioid crisis, there remains a need to elucidate the neural mechanisms underlying opioid use disorder with molecular, temporal and spatial precision using newer circuit manipulation strategies in animal models.

**Objectives:**

We sought to characterize the behavioral effects of chemogenetic manipulations of 5-HT DRN neurons in male and female Tph2-iCre rats.

**Methods:**

Subjects received intra-DRN viral infusions of Cre-dependent inhibitory or excitatory Designer Receptors Exclusively Activated by Designer Drugs (DREADDs). Three behavioral paradigms were used to examine the effect of clozapine-N-oxide activation or inhibition of 5-HT DRN neurons: elevated plus maze (EPM), forced swim test (FST), and stress-induced reinstatement of morphine conditioned place preference (CPP).

**Results:**

Chemogenetic activation of 5-HT DRN neurons induces anxiety-like behavior in the EPM in a transgenic rat model.

**Conclusions:**

This study confirms some of the findings of prior chemo- and optogenetic studies in mice showing a role of 5-HT DRN neurons in anxiety-like behaviors but did not support effects in the FST model or in reinstatement of morphine CPP. Given the growing availability of transgenic rat lines, future studies in transgenic rats are needed to help dissect a complex literature on the behavioral functions of the 5-HT system.

**Supplementary Information:**

The online version contains supplementary material available at https://doi.org/10.1007/s00213-025-06947-z.

## Introduction

Substance use disorder is marked by repeated relapse to drug use, and stress very often triggers relapse, even after prolonged abstinence (Goeders 2003; Sinha 2008). The dorsal raphe nucleus (DRN), which contains most of the forebrain-projecting serotonin (5-hydroxytryptamine, 5-HT) neurons (Jacobs and Azmitia 1992), has been implicated in psychiatric disorders related to stress and substance use disorders as well as their preclinical models (Baldwin and Rudge 1995; Mann 1999; Ettenberg et al. 2011; Staub et al. 2012; Li and Kirby 2016).

Stressors induce a negative affective state that can motivate drug-seeking behavior (Markou 1998; Koob 2010) as modeled in stress-induced reinstatement of previously extinguished conditioned place preference (CPP). Our previous work using morphine CPP indicates that GABAergic inhibition of the DRN induces reinstatement, while disinhibiting the DRN protects against stress-induced reinstatement (Li et al. 2013a). In a subsequent study with the same paradigm, intra-DRN corticotropin-releasing factor receptor 1 (CRF-R1) activation, known to mediate stress-induced inhibition of the 5-HT DRN system (Kirby 2000; Price et al. 2002; Kirby et al. 2008), induced reinstatement, while CRF-R1 antagonism attenuated swim stress-induced reinstatement. Evidence that the latter manipulation reduces negative affective state came from the finding that intra-DRN pretreatment with a CRF-R1 antagonist attenuated foot shock stress-induced 22 kHz ultrasonic vocalizations (USVs) (Li et al. 2021), distress calls that signal negative affect (Barker et al. 2015; Simola and Granon 2019). These findings suggest a causal relationship between 5-HT activity in the DRN and stress-related behaviors.

Both of these earlier studies involved indirect pharmacological manipulation of 5-HT neurons in the DRN. The current study explores the direct activation and inhibition of 5-HT DRN neurons using viral delivery of Cre-dependent Designer Receptors Exclusively Activated by Designer Drugs (DREADDs) in the Tph2-iCre rat (Weber et al. 2011). This chemogenetic technology allows spatial and temporal control over activity in molecularly defined neurons or circuits in vivo (Smith et al. 2021). In mice, Gq activation of 5-HT DRN neurons in 5-HT-targeted Cre driver lines has produced largely anxiogenic effects in multiple models including elevated plus maze (EPM), open field, and light-dark transition (Teissier et al. 2015; Urban et al. 2016), but see (Venner et al. 2020). These studies also found consistent reductions in depression-like behaviors in models including the forced swim and tail suspension tests (Teissier et al. 2015; Urban et al. 2016). Other studies showed similar anxiogenic phenotypes with chemogenetic activation of 5-HT DRN projections to the nucleus accumbens (You et al. 2016) and amygdala (Ren et al. 2018a, b), and antidepressant phenotypes with chemogenetic activation of 5-HT DRN projections to the orbitofrontal cortex (Ren et al. 2018a, b). You et al. (2016) also found that chemogenetic activation of 5-HT DRN neurons projecting to the nucleus accumbens reduced cocaine reward in a CPP paradigm. Chemogenetic inhibition of 5-HT DRN neurons or projections was without effect when tested in these models (Teissier et al. 2015; You et al. 2016). Much less is known in rats as transgenic models have become available only more recently. One group used dual viral strategies in Sprague-Dawley rats to target DRN neurons projecting to the dorsal hippocampus and found that chemogenetic silencing of the pathway attenuates cocaine-seeking behavior in a self-administration paradigm (Kohtz et al. 2024). Another group used a lentivirus to deliver an optogenetic actuator under a Tph2 promotor to control 5-HT DRN neuronal activity in rats (Nishitani et al. 2019). This study showed that optogenetic activation of 5-HT neurons decreases depression-like behaviors in the forced swim test, and inhibition of 5-HT neurons is anxiogenic in the EPM (Nishitani et al. 2019). While the antidepressant phenotype is consistent with chemogenetic studies in transgenic mice (Teissier et al. 2015; Urban et al. 2016), the anxiety phenotype is not, underscoring the need for utilizing the available transgenic tools in both rats and mice to better understand the role of 5-HT in behavioral models.

The Tph2-iCre rat, a transgenic rat line with tamoxifen-inducible Cre recombinase under the control of a Tph2 promotor, was originally developed by Weber et al. for the targeted manipulation of the 5-HT system (Weber et al. 2011). To date, other than in our recent publications in alcohol and heroin self-administration models (McElroy et al. 2025; Li et al. 2024) there do not appear to be any publications behaviorally characterizing the effects of 5-HT neuron manipulation with opto- or chemogenetic tools in this transgenic strain. Here, we use chemogenetic strategies to characterize the behavioral effects of manipulating 5-HT DRN neurons in male and female Tph2-iCre rats using the morphine CPP, EPM, and FST paradigms.

## Methods

### Subjects

Male and female Tph2-iCre rats on a Sprague-Dawley background were developed by Drs. Dusan Bartsch and Kai Schoenig (Weber et al. 2011) and breeders generously provided by Drs. Katinka Stecina and Larry Jordan at the University of Manitoba, Canada. At weaning, rats were housed 2–4/cage with enrichment until 8–9 weeks of age. Hemizygous Tph2-iCre rats and wild-type (WT) littermates received intraDRN AAV injections. Animals were housed under a 12-hour light/dark cycle (lights on at 8:00AM) at 20 °C temperature and 40% humidity. All animal procedures were conducted under protocols approved by the Temple University Institutional Animal Care and Use Committee in accordance with the National Research Council’s *Guide for the Care and Use of Laboratory Animals*.

## IntraDRN AAV injections

Following anesthesia with ketamine/xylazine (100/10 mg/kg, i.m.), rats were positioned in a Narishige stereotaxic apparatus, the skull exposed and hole drilled to lower a Hamilton syringe into the DRN (7.6 mm posterior to Bregma, 3.4 mm lateral to midline and 6.9 mm (females) or 7.4 mm (males) ventral from the skull surface) at a 26° angle to avoid the sagittal sinus (Paxinos and Watson 1998). AAV constructs (AAV8-hSyn-DIO-hM3D(Gq)-mCherry, ≥ 4 × 10^12^ vg/ml or AAV8-hSyn-DIO-hM4D(Gi)-mCherry, ≥ 1 × 10^13^ vg/ml or the empty (control) vector AAV8-hSyn-DIO-mCherry, ≥ 1 × 10^13^ vg/ml; Addgene, Watertown, MA) were infused at a rate of 0.2µL/min over 10 min for a total volume of 2µL, after which the probe remained for an additional 5 min to allow thorough diffusion of the virus through the DRN. Animals received the analgesic ketoprofen (5 mg/kg, s.c.) prior to surgery and on postoperative day 1. Another dose was administered on postoperative day 2 if animals showed signs of pain. Rats were housed singly after surgeries and, following one week of recovery, received daily injections of tamoxifen (40 mg/kg, i.p.; Sigma-Aldrich, St. Louis, MO) for 5 days to activate Cre recombinase. Behavioral testing (separate cohorts for each test described below) began 1–2 weeks after tamoxifen treatment.

Successful viral infusions into DRN 5-HT neurons were confirmed following behavioral testing: for hemizygous Tph2-iCre animals, by fluorescence microscopic observation of robust mCherry expression confined to the DRN; for WT littermates, by histological observation of the DRN cannula track and absence of tissue damage. To more systematically quantify viral expression in the DRN, we developed a semi-automated pipeline which is described in the Supplemental Methods section. While there is no standardized threshold for the count of labeled cells that indicates successful viral expression, a few precedents in the chemogenetics literature can guide establishment of a cutoff: Aery Jones, et al. (2021) excluded an animal for having fewer than 50 mCherry + cells; McCosh et al. (2024); excluded animals with fewer than 5 mCherry + cells; and Radzicki, et al. (2025) excluded animals that had no visible expression. We decided that an animal would meet inclusion criteria if across all available slices (images) the count of mCherry + cells totaled at least 100. Supplemental Fig. 1 displays representative images of mCherry expression level for each viral group with atlas overlay, and for comparison, an image of expression that did not meet inclusion criteria.

Using these inclusion criteria, of 153 subjects tested in EPM experiments, 29 were histologically excluded from data analysis. Of 32 subjects tested in FST experiments, 6 were histologically excluded from data analysis. Of 112 subjects tested in CPP experiments that passed behavioral criteria (see below), 36 were histologically excluded from data analysis. For these chemogenetic studies, the experimental group was composed of hemizygous Tph2-iCre rats with intraDRN AAV delivery of Cre-dependent Gq or Gi DREADDs followed by tamoxifen induction and CNO injection during the behavioral test. Control subjects included WT controls (WT littermates with intraDRN DREADDs, tamoxifen induction and CNO during the test), viral controls (hemizygous Tph2-iCre rats with intraDRN control AAV, tamoxifen induction and CNO during the test), and vehicle controls (hemizygous Tph2-iCre rats with intraDRN DREADDs, tamoxifen induction and vehicle injection during the test), as side effects from CNO metabolism have been shown to variably impact behavior, even at relatively low doses typical in DREADDs experiments (MacLaren et al. 2016; Ilg et al. 2018). See Table 1 for counts in each group. Behavioral experiments were run on separate cohorts (of 8–12 rats). Animals tested in FST were exposed to that single protocol. As EPM is a less disruptive protocol, one week after EPM exposure, the same animals then underwent CPP testing (except for three cohorts, which underwent only CPP testing).

**Table 1:** Subject count in each experimental and control group

				*EPM*	*FST*	*CPP*	
**Genotype**	**DREADDs**	**Tx**	**Sex**	**-**	**-**	**Swim**	**No-Swim**
Hemi	Gq	CNO	M	12	3	8	NA
			F	8	4	8	NA
		Veh	M	4	NA	3	NA
			F	1	NA	0	NA
	Gi	CNO	M	11	5	NA	8
			F	14	4	NA	4
		Veh	M	3	NA	NA	1
			F	1	NA	NA	0
	mCherry	CNO	M	3	5	1	0
			F	4	5	0	3
WT	Gq	CNO	M	9	NA	10	NA
			F	14	NA	9	NA
		Veh	M	10	NA	4	NA
			F	4	NA	1	NA
	Gi	CNO	M	9	NA	NA	6
			F	14	NA	NA	6
		Veh	M	1	NA	NA	4
			F	2	NA	NA	0

Regarding chemogenetic efficacy, we showed in a recent publication from our laboratory (Li et al. 2024; Fig. 2) that following tamoxifen induction, there was nearly 100% colocalization of Cre and TPH2-IR + in the DRN of Tph2-iCre rats. We also showed that following viral delivery of Cre-dependent DREADDs and tamoxifen induction, robust DRN Cre expression was observed in Tph2-iCre rats but not their WT littermates. Lastly, we provided electrophysiological evidence that activation of Gq DREADDs in these animals depolarizes 5-HT DRN neurons whereas activation of Gi-DREADDs hyperpolarizes 5-HT DRN neurons (Li et al. 2024; Fig. 2). Collectively, these data validate the Tph2-iCre rat strain and demonstrate functionality of DREADD activation on 5-HT DRN neuronal activity at the single-cell level. These previous validations in combination with behavioral effects of DREADDs in this study and other studies in our laboratory (EtOH self-administration: McElroy et al. 2025; heroin self-administration: Li et al. 2024) give us confidence that DREADDs are being successfully engaged in our protocol to modulate 5-HT neurotransmission in a physiologically relevant manner.

## Elevated plus maze (EPM)

For EPM testing, the DREADD agonist clozapine N-oxide (CNO; 2 mg/kg, i.p.) or saline was injected 30 min prior to placing the animal in an open arm of the EPM apparatus facing the center in a red-lit room. The 50-cm-high custom Plexiglas apparatus consisted of two open arms (28 × 7 cm), with a 0.5-cm lip on each open arm, and two enclosed arms (30 × 7 × 13.5 cm) that extended from a central platform (7 × 7 cm). Behavior was videotaped for 5 min. Time and number of entries into the open arms, closed arms and center were recorded (see Discussion for further details). Open arm time and open arm entries are sensitive to anxiolytic treatments in the EPM (Hogg 1996).

## Forced swim test (FST)

Rats were first exposed to a 15-min pretest swim in a 20-cm wide cylindrical swim tank filled with room-temperature (21–22 °C) water to a depth of 30 cm followed 24 h later by a 5-min test swim in which behavior was videotaped (Porsolt et al. 1978). CNO (2 mg/kg, i.p.) was administered in a subchronic regimen: three times, at 23.5, 4.5 and 1 h prior to the swim test. One of four behaviors was scored at 5-min intervals during the test swim (Detke et al. 1995): immobility (minimum movement necessary to stay afloat), swimming (active swimming across quadrants of the swim tank), climbing (vigorous movements of the forepaws directed against the side of the tank) and diving. The FST is considered an antidepressant screen as multiple classes of antidepressants administered in this subchronic regimen reduce passive coping responses (immobility) and increase active coping responses (swimming, climbing) to the inescapable 5-min swim stress (Cryan et al. 2005).

## Morphine conditioned place-preference (CPP), extinction and stress-induced reinstatement

The CPP procedure was an unbiased design, conducted in low-light (max 125 lx) conditions with 20 × 20 × 40-cm custom Plexiglas chambers consisting of two compartments separated by a removable partition. While equally sized, the separate compartments differed in wall pattern (horizontal vs. vertical black stripes on white background), lighting (unlit vs. illuminated by a small spotlight), and floor color and texture (smooth black surface vs. wire mesh over white surface). Preconditioning testing ensured no preference by the group for either compartment (average difference of time spent on each side  < 100 s over a 15-min free-access test; Li et al. 2021). The morphine dosage of 5 mg/kg dissolved in 0.9% saline was selected for its ability to induce robust CPP that could be extinguished and then reinstated by swim stress (Staub et al. 2012b; Li et al. 2013).

### Conditioning

For the first four days of the CPP protocol, morphine or saline was administered subcutaneously in the morning (10am) or afternoon (3pm) in an alternating fashion before confining the animal to a compartment for 45 min. The drug-paired compartment and the drug order for each animal was randomized across the group. A conditioning test on day 5 consisting of 15 min of free access to both compartments confirmed morphine CPP if a difference of at least 100 s was observed in time spent in the drug-paired side minus that spent in the saline-paired side (Li et al. 2021). Of 164 subjects, 23 failed to meet conditioning criteria and were excluded from the study.

### Extinction

The first 2 days of the extinction phase (days 6 and 7) consisted of saline injection and confinement to alternating chambers. The second 2 days of the extinction phase (days 8 and 9) consisted of no injections and confinement to alternating chambers. An extinction test on day 10 consisted of a 15-min free-access session. Extinction was confirmed if a difference of less than 100 s was observed in time spent in the drug-paired side minus that spent in the saline-paired side (Li et al. 2021). Animals failing to meet extinction criteria were given additional no-injection extinction training sessions followed by an extinction test on the subsequent day until they reached extinction criteria. Animals that failed 4 extinction tests were removed from the experiment. Animals passing the extinction test were given a stress-induced reinstatement test the following day. Of 141 subjects that passed conditioning criteria, 29 failed to meet extinction criteria and were excluded from the study.

### Stress-induced reinstatement

For stress-induced reinstatement, animals were first exposed to swim stress: placement into a 20-cm wide cylindrical swim tank filled with room-temperature (21–22 °C) water to a depth of 30 cm for 5 min. Animals were then towel-dried and placed into a warming cage for 20 min before exposure to a 15-min free-access reinstatement test.

### Experimental design

To test the hypothesis that excitation of serotonergic DRN neurons protects against stress-induced reinstatement to morphine CPP, animals with intraDRN viral delivery of excitatory Gq DREADDs received an intraperitoneal injection of CNO (2 mg/kg, i.p.) or saline 30 min prior to forced swim stress-induced reinstatement. To test the hypothesis that inhibition of 5-HT DRN neurons independently induces reinstatement to morphine CPP in the absence of stress, animals with intraDRN viral delivery of inhibitory Gi DREADDs received an intraperitoneal injection of CNO (2 mg/kg, i.p.) or saline 30 min prior to a reinstatement test without prior swim stress exposure.

### Data analysis

Animals were included in data analysis if they met histology criteria for successful viral injections and behavioral criteria as described above. R version 4.3.2 (https://www.r-project.org/) was used for statistical analyses and GraphPad Prism 10 (Dotmatics, San Diego, CA) for graphics. Data were tested for normality and equal variance prior to statistical testing and reported as mean ± SEM. All groups passed Levene’s test for homoscedasticity. Normality was violated for some groups in CPP and Gi-EPM, but non-parametric PERMANOVAs converged on ANOVA results. No ANOVA assumptions were violated for the Gq-EPM group reporting significant results. One-way ANOVA tests were conducted to justify pooling control groups: no effects were found for any metric (detailed below) in any experiments, so controls were analyzed as a pooled male and pooled female group for each behavioral test. For EPM experiments, open arm entries and % time in open arms were analyzed by 2-way factorial ANOVA with sex and virus (mCherry control vs. either Gq or Gi DREADD) as between-subjects factors. Animals receiving the control mCherry virus (*N* = 7) functioned as controls in both the Gi and Gq EPM analyses (see Discussion). For FST experiments, mean immobility, swimming or climbing counts were analyzed across experimental groups by 2-way factorial ANOVA with sex and virus (control, Gq, Gi DREADD) as between-subjects factors. FST data were further examined by collapsing across viral treatment groups or across sex to look more closely at sex differences or virus effects, respectively.

In the no-swim CPP experiment, a subset of female WT Gi-infused CNO-treated controls (*N* = 7 across three cohorts) displayed unexpected reinstatement behavior (6 of 7 reinstated). Because this pattern was not observed in male counterparts, it raised concerns about unknown external factors, possibly including batch effects from the in-house bred Tph2-iCre line, sex differences in reactivity to injection stress, and variable influence of CNO and its metabolites on 5-HT and DA systems (Griebel et al. 1997; Natesan et al. 2007; Ilg et al. 2018). To test if this pattern persisted under identical conditions, we ran an additional cohort of female Gi-infused CNO-treated animals (*N* = 12, 6 Hemizygous, 6 WT controls) and observed no unusual reinstatement behavior. Statistical analyses conducted with and without the original subset of female Gi-infused animals (*N* = 18, 10 Hemizygous-CNO, 7 WT-CNO, 1 WT-Veh) yielded equivalent results with no differences in significance, confirming that their inclusion has no impact on data interpretation. Because unknown experimental factors influenced the behavior of the original cohort, and the validation cohort did not replicate that pattern, we excluded the original cohort to ensure the reliability of group comparisons in the final analysis. Future studies could further mitigate potential confounds of stress reactivity and CNO metabolism by employing within-subject designs comparing behavioral effects of CNO vs. vehicle injections (Manvich et al. 2018). For the swim and no-swim CPP experiments, time spent in the drug-paired minus that spent in the unpaired side was compared across experimental groups by 3-way repeated measure ANOVA with sex and virus as between-subjects factors and CPP phase (extinction and reinstatement) as a within-subjects factor.

## Results

**Fig. 1:**
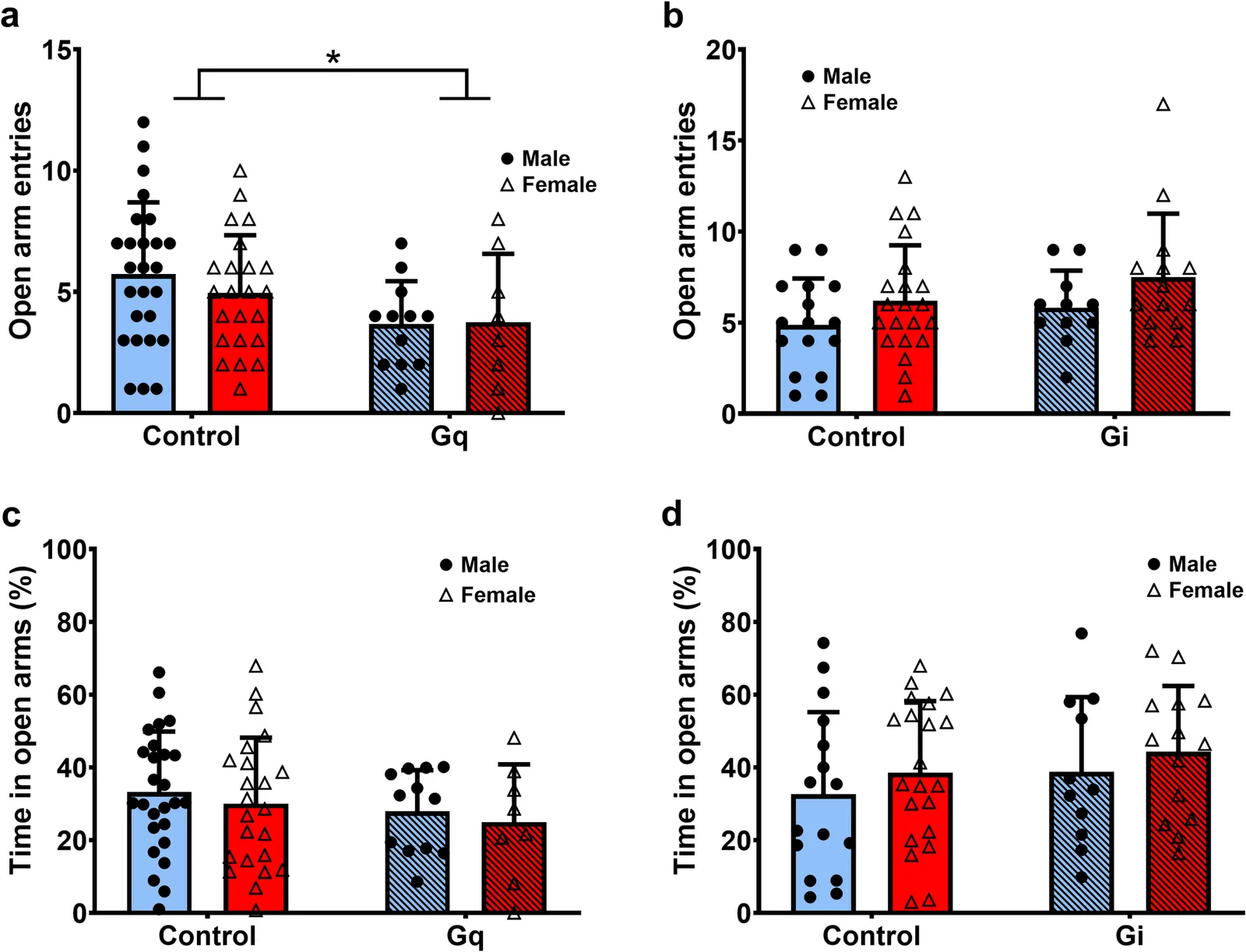
Anxiogenic effect of chemogenetic activation of 5-HT DRN neurons in the EPM Open arm entries (**a**) but not time in open arms (**b**) were significantly reduced in the EPM following chemogenetic activation (Gq) of 5-HT DRN neurons. Chemogenetic inhibition (Gi) of 5-HT DRN neurons had no effect on either open arm entries (**c**) or time in open arms (**d**) in the EPM. No sex differences were observed in the EPM in response to chemogenetic manipulations. * *p* < 0.05, main effect of Gq in 2-way ANOVA. All data are presented as means + SEM error bars. Figures 1a and 1b: male control: N = 26; female control: N = 23; male Gq: N = 12; female Gq: N = 8. Figures 1c and 1d: male control: N = 16; female control: N = 21; male Gi: N = 11; female Gi: N = 14

Figure 1 shows the effects of chemogenetic excitation (Fig. 1a and b, *N* = 69) or inhibition (Fig. 1c and d, *N* = 62) of 5-HT DRN neurons on anxiety-like behaviors in the EPM. Figure 1a shows that Gq activation of 5-HT DRN neurons was anxiogenic, reducing open arm entries [F(1,65) = 5.52, *p* < 0.05], but there was no main effect of sex [F (1, 65) = 0.25, *p* > 0.05] or Gq x sex interaction [F (1, 65) = 0.38, *p* > 0.05]. By contrast, Fig. 1b shows that % time in open arms was unaffected by Gq [F (1, 65) = 1.37, *p* > 0.05] and sex [F (1, 65) = 0.49, *p* > 0.05] and there was no Gq x sex interaction [F (1, 65) = 0.00, *p* > 0.05]. Figure 1c and d show no effects of Gi inhibition of 5-HT DRN neurons on any EPM behaviors. For open arm entries in Fig. 1c there was no effect of Gi [F (1, 58) = 2.23, *p* > 0.05], sex [F (1, 58) = 3.95, *p* > 0.05] or Gi x sex interaction [F (1, 58) = 0.001, *p* > 0.05]. For % time in open arms in Fig. 1d there was no effect of Gi [F (1, 58) = 1.26, *p* > 0.05], sex [F (1, 58) = 1.18, *p* > 0.05] or Gi x sex interaction [F (1, 58) = 0.00, *p* > 0.05].

Data from a subset of Gq- and Gi-treated animals (*N* = 7 and *N* = 21, respectively, collapsed across sex; data not shown) were analyzed using closed and total arm entries as an index of locomotor activity (Hogg 1996; Braun et al. 2012). The Gq activation group (hemizygous Tph2-iCre, Gq, CNO) showed 8.8 ± 1.0 closed arm entries and 13.8 ± 2.1 total arm entries compared to Gq WT controls: 9.0 ± 1.0 closed arm entries and 14.0 ± 1.0 total arm entries. The Gi inhibition group (hemizygous Tph2-iCre, Gi, CNO) showed 8.9 ± 0.9 closed arm entries and 16.0 ± 2.0 total arm entries compared to Gi WT controls: 8.9 ± 0.8 closed arm entries and 14.9 ± 0.9 total arm entries. There were no differences by unpaired t-test between experimental and control groups in either the Gq or Gi group for either closed or total arm entries, indicating no differences in motor activity in response to either stimulation or inhibition of 5-HT DRN neurons.

**Fig. 2:**
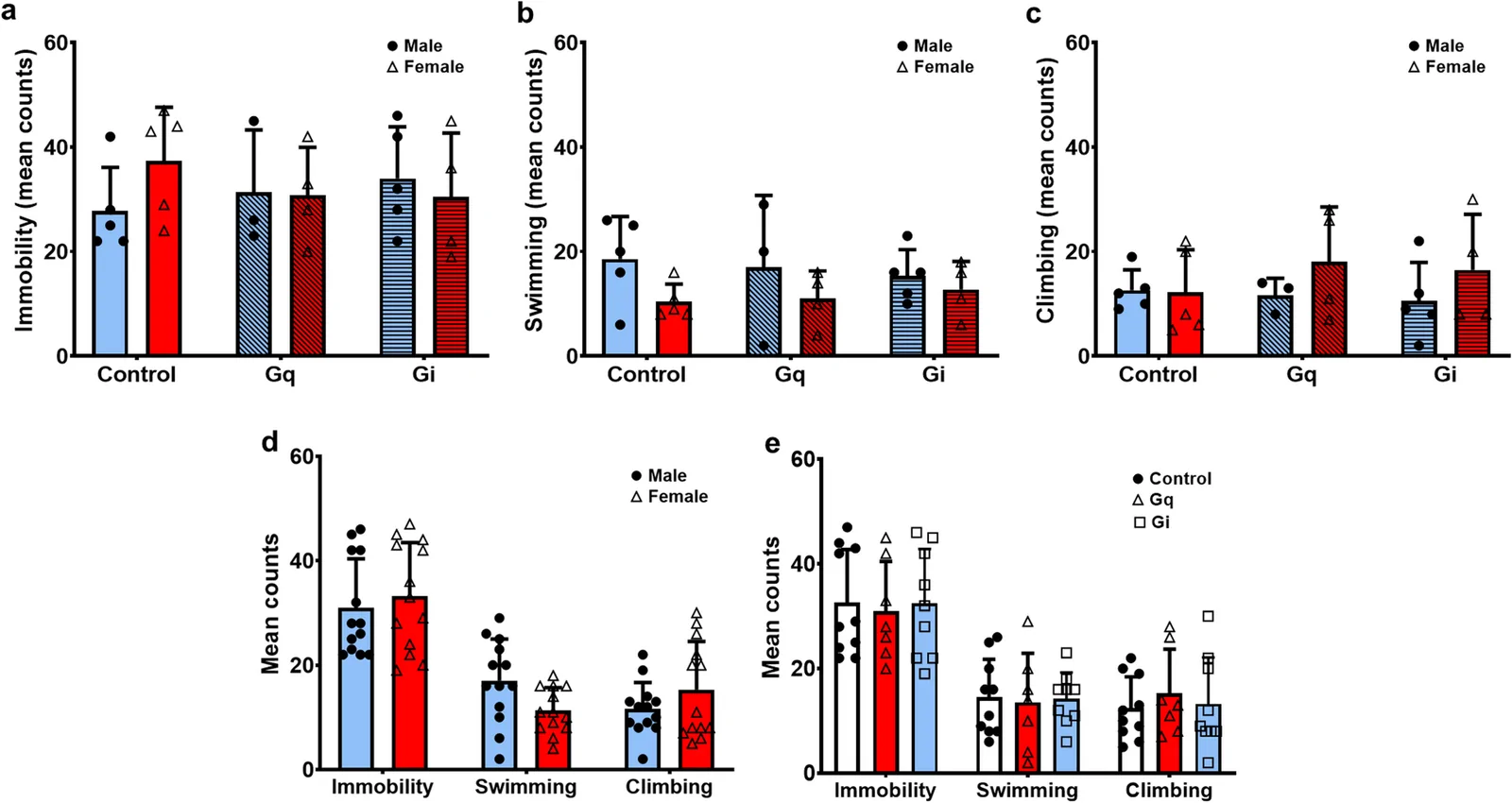
No effect of chemogenetic manipulation of 5-HT DRN neurons in the FST. There were no effects of either chemogenetic activation (Gq) or inhibition (Gi) of 5-HT DRN neurons on immobility (**a**), swimming (**b**) or climbing (**c**) behaviors in the FST. When subjects were pooled across chemogenetic treatment groups, no sex differences were observed on any of the three behavioral endpoints in the FST (**d**). When males and females were pooled, there were no effects of chemogenetic manipulations on any of the three behavioral endpoints in the FST (**e**). All data are presented as means + SEM error bars. Figures 2a-2c: male control: N = 5; female control: N = 5; male Gq: N = 3; female Gq: N = 4; male Gi: N = 5; female Gi: N = 4. Figure 2d (for each behavior): male: N = 13; female: N = 13. Figure 2e (for each behavior): mCherry control: N = 10; Gq: N = 7; Gi: N = 9.

Figure 2 shows the effects of chemogenetic excitation or inhibition of 5-HT DRN neurons in the FST (*N* = 26). Figure 2a shows that immobility was not affected by DREADDs [F (2, 20) = 0.04998, *p* > 0.05], sex [F (1, 20) = 0.2051, *p* > 0.05], or DREADDs x sex interaction [F (2, 20) = 1.073, *p* > 0.05]. Figure 2b shows that swimming was not affected by DREADDs [F (2, 20) = 0.01362, *p* > 0.05], sex [F (1, 20) = 4.143, *p* > 0.05], or DREADDs x sex interaction [F (2, 20) = 0.3812, *p* > 0.05]. Figure 2c shows that climbing was not affected by DREADDs [F (2, 20) = 0.1963, *p* > 0.05], sex [F (1, 20) = 1.582, *p* > 0.05], or DREADDs x sex interaction [F (2, 20) = 0.5228, *p* > 0.05]. Figure 2d shows that FST behavior was not affected by sex [F (1, 84) = 0.002820, *p* > 0.05] or behavior x sex interaction [F (2, 84) = 0.7183, *p* > 0.05]. There was a main effect of behavior [F (2, 84) = 36.07, *p* < 0.0001]. Figure 2e shows that FST behavior was not affected by DREADDs [F (2, 69) = 0.002035, *p* > 0.05] or behavior x DREADDs interaction [F (4, 69) = 0.1771, *p* > 0.05]. There was a main effect of behavior [F (2, 69) = 39.23, *p* < 0.0001].

**Fig. 3:**
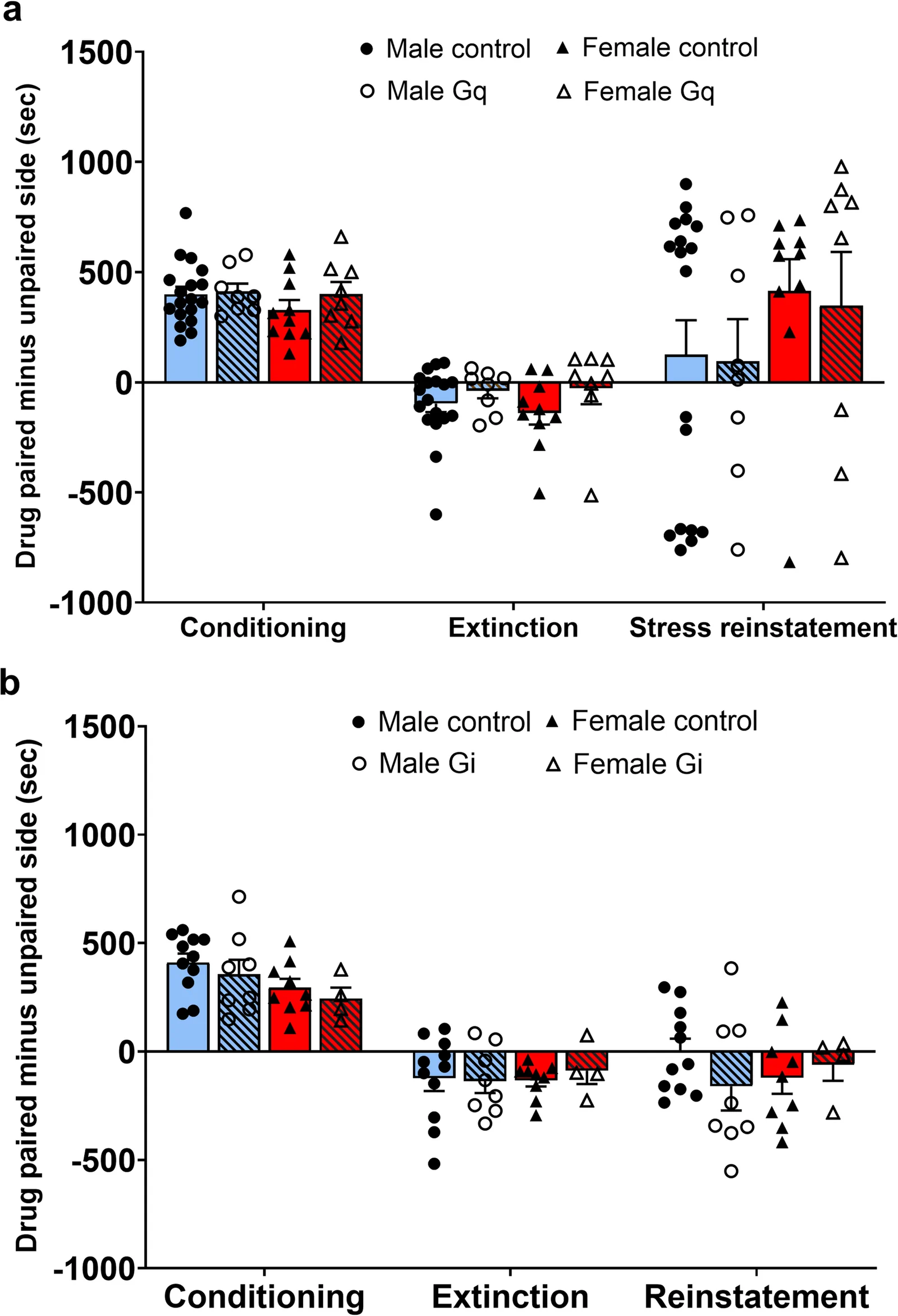
No effect of chemogenetic manipulations of 5-HT DRN neurons in reinstatement of morphine CPP. Chemogenetic activation (Gq) of 5-HT DRN neurons had no effect on stress-induced reinstatement (**a**). Chemogenetic inhibition (Gi) of 5-HT DRN neurons did not reinstate previously extinguished morphine CPP (**b**). All data are presented as means ± SEM error bars. 3a male control: N = 18, female control: N = 10, male Gq: N = 8, female Gq: N = 8. 3b male control: N = 11, female control: N = 9, male Gi: N = 8, female Gi: N = 4.

Figure 3 shows the effects of chemogenetic excitation or inhibition of 5-HT DRN neurons on reinstatement of previously extinguished morphine CPP. Figure 3a (*N* = 44) shows that there was a main effect of reinstatement [F (1, 40) = 9.13, *p* < 0.01], but no effects of sex [F (1, 40) = 1.85, *p* > 0.05], Gq [F (1, 40) = 0.04, *p* > 0.05], reinstatement x sex interaction [F (1, 40) = 1.82, *p* > 0.05], reinstatement x Gq interaction [F (1, 40) = 0.39, *p* > 0.05], sex x Gq interaction [F (1, 40) = 0.00, *p* > 0.05], or reinstatement x sex x Gq interaction [F (1, 40) = 0.05, *p* > 0.05]. Figure 3b (*N* = 32) shows no effects of reinstatement [F (1, 28) = 0.68, *p* > 0.05], sex [F (1, 28) = 0.00, *p* > 0.05], Gi [F (1, 28) = 0.08, *p* > 0.05], reinstatement x sex interaction [F (1, 28) = 0.12, *p* > 0.05], reinstatement x Gi interaction [F (1, 28) = 0.61, *p* > 0.05], sex x Gi interaction [F (1, 28) = 1.29, *p* > 0.05], or reinstatement x sex x Gi interaction [F (1, 28) = 0.91, *p* > 0.05]. These data indicate stress-induced reinstatement in Fig. 3a and no difference between extinction and reinstatement in the absence of a stressor in Fig. 3b, but no effects of chemogenetic manipulations on reinstatement in either experiment.

## Discussion

This study demonstrated that chemogenetic activation of 5-HT DRN neuronal activity in Tph2-iCre rats increases anxiety-like behavior in the elevate plus maze model. Though there is a substantial literature in 5-HT-specific Cre mouse lines (Hainer et al. 2015) that have probed the behavioral role of the 5-HT system with chemo- and optogenetic strategies, this study is the first to use a 5-HT-specific Cre rat line to examine the role of the 5-HT system in anxiety- and depression-like behaviors. This Tph2-iCre rat line was developed in the laboratory of Dr. Dusan Bartsch (Weber et al. 2011) as a tool to explore the function of the 5-HT system with Cre-dependent viral strategies in a species with a rich behavioral repertoire that has been the basis of much of the existing 5-HT behavioral pharmacology and physiology literature to date. This inducible Cre rat line enables future researchers to use circuit monitoring and manipulation technologies to probe 5-HT neuronal activity in vivo with molecular, spatial and temporal precision.

The anxiogenic phenotype that we observed in rats recapitulates similar anxiogenic phenotypes in several behavioral models following chemo- or optogenetic activation of 5-HT DRN neurons in multiple mouse 5-HT-specific Cre lines including Pet1-Cre (Teissier et al. 2015) and SERT-Cre (Urban et al. 2016), and following activation of DRN projection-specific 5-HT neurons including those terminating in the bed nucleus of the stria terminalis (Marcinkiewcz et al. 2016), amygdala (Ren et al. 2018a, b) and reticulotegmental nucleus (Guo et al. 2022). An additional study in Wistar rats that used intersectional viral strategies and optogenetics to stimulate 5-HT DRN neurons projecting to the basolateral amygdala found similar elevations of anxiety-like behaviors in the social interaction and conditioned fear tests (Bernabe et al. 2020). Other Cre mouse studies have found either no effect or anxiolytic phenotypes following activation of all 5-HT DRN neurons (Correia et al. 2017; Ohmura et al. 2014) or specific projection populations including those to the orbitofrontal (Ramkumar et al. 2024; Ren et al. 2018a, b) and medial prefrontal cortex (Morgan 2023). The diversity of these responses is likely driven by differences including methodology (chemo- vs. optogenetics), Cre driver strain, the anxiety model employed (Venner et al. 2020), and the efferent connectivity of the 5-HT population that is activated (Ren et al. 2018a, b).

Interestingly, chemogenetic inhibition of 5-HT DRN neurons did not produce an anxiolytic phenotype in Tph2-iCre rats, as has been shown in some (Guo et al. 2022; Zhang et al. 2020) but not all of the 5-HT-specific Cre mouse literature (Garcia-Garcia et al. 2018; Morgan 2023; Teissier et al. 2015). It is possible that the acute nature of the chemogenetic inhibition was insufficient to impact the behavioral endpoints in our study (anxiety- and depression-like behaviors as well as opioid reinstatement), and that a more chronic inhibition might be required to produce a measurable behavioral phenotype that includes anxiolysis, as is the case in mice with constitutive gene manipulations that suppress the 5-HT system across the lifespan (Kim et al. 2009; Kiyasova et al. 2011; Mosienko et al. 2012; Narboux-Nême et al. 2011). Urban et al. (2016) used DREADD-assisted metabolic mapping (DREAMM) techniques to show that there were broader changes in metabolic activity of DRN targets following chronic chemogenetic DRN manipulations than following acute manipulations. Others have shown that the effects of these manipulations are only revealed when the system is dysregulated: Teissier et al. (2015) showed that acute chemogenetic inhibition of 5-HT DRN neurons in Pet1-Cre mice was ineffective in naïve subjects but blunted the anxiety phenotype of mice that were previously exposed to postnatal fluoxetine treatment. These authors suggest that this early developmental intervention creates a 5-HT imbalance which reveals the behavioral impact of the chemogenetic inhibition (Teissier et al. 2015). Similarly, Zhang et al. (2020) demonstrated that chemogenetic inhibition of 5-HT DRN neurons in SERT-Cre mice reverses the anxiety-like phenotype of mice with additional knockdown of the receptor tyrosine kinase gene ErbB4 in 5-HT DRN neurons, a manipulation that elevates baseline 5-HT DRN excitability as well as anxiety.

Our study also found that chemogenetic manipulations of 5-HT DRN neurons had no effect in the FST model of depression-like behavior. These FST data differ from the Cre mouse literature which has largely shown antidepressant-like effects in the FST or tail suspension test following chemo- or optogenetic stimulation of 5-HT DRN neurons (Ohmura et al. 2020; Teissier et al. 2015; Urban et al. 2016) or projection-specific 5-HT DRN populations including those to the nucleus accumbens (You et al. 2016) and orbitofrontal cortex (Ren et al. 2018a, b). Additional studies in conventional rat strains that used optogenetics with intersectional viral strategies to stimulate the DRN-lateral habenula circuit (Zhang et al. 2018) or with targeted lentiviruses to stimulate 5-HT DRN neurons (Nishitani et al. 2019) found similar reduced depression-like behaviors in the FST (Nishitani et al. 2019; Zhang et al. 2018) and sucrose preference test (Zhang et al. 2018). Interestingly, Zhang et al. (2018) only observed this antidepressant phenotype in rats exposed previously to a chronic unpredictable mild stress model of depression (Zhang et al. 2018). It is therefore possible that the lack of effect of manipulation of 5-HT neurons in the FST in our study reflects a particular sensitivity of rats to the baseline depression-like and/or serotonergic phenotype, compared to mice.

For this study we were targeting the entire DRN for chemogenetic manipulation. A limitation of this approach is that, given the known functional topography of the DRN and its subdivisions (Lowry et al. 1996), we may have activated or inhibited subdivisions with opposing effects on our behavioral endpoints, thus resulting in a null net effect. For example, Paul and Lowry (2013) showed that dorsal/caudal DR (DRD/DRC) and the ventrolateral “wings” (DRVL/VLPAG) contribute differentially to anxiety-related responses; and Ren, et al. (2019) showed that amygdala- vs. frontal-cortex-projecting DR 5-HT neurons can have divergent effects on anxiety-related behavior. In future studies we could reduce the viral injection volume to target specific subregions.

Our observation of a significant difference in open-arm entries for the Gq EPM study (*p* = 0.022) appears to contrast with the null effect in percent time in open arms, as well as the null effects in the Gi group. While mean fluorescent cell dispersion between Gq and Gi groups did not significantly differ (*p* = 0.13, see Supplemental Methods), our results might be explained by slight variations in viral spread across DRN subregions that could shift EPM phenotypes. Another consideration is the fact that our primary analysis revealing the anxiety phenotype in animals with chemogenetic activation of 5-HT DRN neurons was conducted with a pooled set of controls. To confirm the phenotype, we also compared experimental animals (hemizygous, Gq, CNO) to the WT control group (WT, Gq, CNO) which controls for the potential off-target effects of CNO administration (Ilg et al. 2018). This comparison also revealed the same findings of an anxiety-like phenotype (increased open arm entries) in the experimental group.

In the EPM, examining percent open-arm entries (open/total) and a locomotor proxy (total or closed-arm entries) could potentially help distinguish avoidance from hypoactivity (Braun et al. 2012). For strong anxiety phenotypes, decreased open arm entries often tracks with decreased open arm time. As an effect was found in one but not the other of those endpoints, the phenotype seems to be subtle in the Gq-treated animals. However, to distinguish this effect from a general locomotion phenotype, data from a subset of Gq- and Gi-treated animals (*N* = 7 and *N* = 21, respectively) were analyzed with closed and total arm entries as an index of locomotor activity (Hogg 1996; Braun et al. 2012). Because initial analysis of these data showed no sex differences in any experimental or control group for either closed or total arm entries, the data were collapsed across sex to produce four groups. There were no differences by unpaired t-test between experimental and control groups in either the Gq or Gi group for either closed or total arm entries, indicating no differences in motor activity in response to either stimulation or inhibition of 5-HT DRN neurons. Measuring a greater variety of locomotor proxies, including number of rears and head dips, for more animals would be an improvement in future EPM studies. While the FST is primarily sensitive to antidepressant drug treatment, it is also sensitive to manipulations that impact motor activity. As a consequence, positive FST results should be followed by independent tests of locomotion to rule out motor confounds to these data (Lucki 1997). Therefore, the lack of effect of our chemogenetic manipulations in the FST is further support that locomotor activity is unaffected in these animals. We do acknowledge, however, the small sample sizes for the FST experiment (*N* = 3–5 grouped by sex and virus).

A number of studies in the Cre mouse literature have shown a role for 5-HT DRN neurons in reward. Several studies have shown optogenetic stimulation of 5-HT DRN neurons to be rewarding (Fu et al. 2022; Liu et al. 2014; Nagai et al. 2020), though this effect appears to be mediated in part by co-released glutamate (Liu et al. 2014, 2020; Qi et al. 2014). Others have shown that 5-HT DRN stimulation promotes waiting for reward (Fonseca et al. 2015; Miyazaki et al. 2014), rather than being intrinsically rewarding (Fonseca et al. 2015). When these manipulations were tested in models of drug or natural reward, the results have been mixed. Simulation of 5-HT DRN neurons reduces cocaine CPP (You et al. 2016) but has no effect on operant responding for saccharin (Browne et al. 2019), whereas inhibition of 5-HT DRN neurons can suppress morphine CPP (Fu et al. 2022). Recent studies in our laboratory in the Tph2-iCre rat strain have shown that chemogenetic activation of 5-HT DRN neurons suppresses alcohol and sucrose self-administration (McElroy et al. 2025) but elevates heroin self-administration (Li et al. 2024), though these effects may reflect a leftward shift in the dose-response curve across these natural and drug rewards (McElroy et al. 2025), potentially indicating an overall reduction in reward value. These studies also showed that punished responding for both alcohol and heroin is elevated by chemogenetic activation of DRN 5-HT neurons, indicating that this manipulation promotes punishment-resistant (i.e. compulsive) drug consumption (McElroy et al. 2025; Li et al. 2024). In contrast, the current study found no effect of chemogenetic manipulations on 5-HT DRN neurons in reinstatement of previously extinguished morphine CPP, a model of opioid relapse. No Cre mouse studies to date appear to have tested the effect of opto- or chemogenetic 5-HT manipulations in reinstatement models. So, while it appears that opioid reward is sensitive to chemogenetic manipulations of the 5-HT DRN system (Fu et al. 2022; Li et al. 2024), our current data in rats would suggest a lack of sensitivity of opioid reinstatement models to these manipulations.

In summary, the current study confirms some of the findings of prior chemo- and optogenetic studies in mice for a role of 5-HT DRN neurons in anxiety-like behaviors but did not support effects in the FST model or in reinstatement of morphine CPP. With the availability of this 5-HT-specific Cre rat line and a growing availability of transgenic rat lines, there is a clear need for future studies in transgenic rats to help dissect a complex literature on the behavioral functions of the 5-HT system. These future studies will also benefit from more select targeting of 5-HT DRN subpopulations with distinct afferent and efferent connectivity, from direct comparison of acute vs. chronic manipulations of the system as well as examination of the effects of such manipulations under conditions of 5-HT dysregulation.

## Supplementary Information

Below is the link to the electronic supplementary material.

Supplementary File 1 (DOCX 1.55 MB)

## Data Availability

The raw data that support the findings of this study are available to other investigators upon request.
