# Supplementary material for: Behavioral impact of chemogenetic manipulations of 5-HT DRN neurons in transgenic Tph2-iCre rats
Source: Psychopharmacology (Berl). 2025 Nov 15;243(9):2029–41. doi: 10.1007/s00213-025-06947-z (PMC12958828; doi:10.1007/s00213-025-06947-z)
Supplement: Supplementary file 1 [file 213_2025_6947_MOESM1_ESM.docx]

Supplemental Methods

*DREADD quantification*

To more systematically quantify DREADD expression in the DRN, we developed a semi-automated pipeline integrating ImageJ (Schindelin et al., 2012) and Cellpose, an open-source deep-learning model for cellular segmentation (Pachitariu, et al., 2025). First, using the Fiji software, masks delineating the boundaries of the DRN were drawn by hand for each image, and cells exhibiting sufficient expression (i.e., bright mCherry+ somas) were manually counted on 10% of those images (except for mCherry controls, which were proportionally overrepresented due to smaller group size) to establish reference values for calibration.

A pre-trained Cellpose model for cellular segmentation was then used to count cells within the mask-defined region of interest (ROI) in those histology images. Iterative manual and automatic (minimizing mean average percent error on the 10% subset of manual counts) calibration fine-tuned the adjustable parameters (including minimum size in pixels for segmentation and cell detection threshold) to meet the pre-established criterion of 75% agreement. Finally, using the optimized parameters, the model was applied to automatically count cells and compute coverage and dispersion metrics (see below) for the remaining images. All Python scripts for leveraging Cellpose, calibrating its adjustable parameters, and conducting pairwise comparisons (Supplemental Tables 1 & 2), along with further details, are available in the associated GitHub repository: <https://github.com/nmccloskey/DREADDFUL>.

This semi-automated pipeline was applied to images from animals which had already passed qualitative histological assessment (see Methods). After cell counts were totaled across available slices for each animal, those with fewer than 100 total cells were manually reviewed. Following automated cell counting and manual confirmation, a total of 4 additional rats were removed from the EPM/CPP group. Supplemental Figure 1 illustrates representative examples of viral expression in the three viral treatment groups.

**Fig 1. Representative images of DREADDs mCherry expression in the DRN with atlas overlay.** Select histology samples depict expression level meeting inclusion criteria for the (a) Gq, (b) Gi, and (c) mCherry virus groups, plus, for comparison, an example of (d) insufficient expression that fails to meet inclusion criteria. A Paxinos and Watson (1998) atlas overlay is included to identify the DRN and its subregions (DRD: dorsal raphe dorsal, DRV: dorsal raphe ventral, DRVL: dorsal raphe ventrolateral).

*DREADD expression comparison across viral groups*

To investigate systematic differences in expression between viral groups, we conducted two-sided pairwise comparisons (α = 0.05) for each automated expression metric: total cell count (summed across available slices), mean coverage (the proportion of ROI pixels occupied by any segmented cell label), and mean dispersion (the area of the convex hull enclosing the centroids of all segmented cells, normalized by ROI area). For each metric, if the viral groups met criteria for normality (Shapiro-Wilk, α = 0.05) and homoscedasticity (Levene, α = 0.05), comparisons were performed using pooled, two-sample t-tests with Hedges’ g effect sizes; otherwise, using two-sided Mann-Whitney U tests with Cliff’s delta effect sizes. In the “Effect” columns below, positive values indicate that Group1 > Group2 for that metric; and negative values, the reverse.

Holm-Bonferroni family-wise error rate (FWER) and Benjamini–Hochberg false discovery rate (FDR) adjustments were computed across the sets of pairwise comparisons within the EPM/CPP and FST groups (α = 0.05). For the animals run in EPM/CPP experiments (Supplemental Table 1), no differences survived either correction, indicating equivalence of DREADD expression metrics across viral groups. For the FST animals (Supplemental Table 2), the mCherry control group showed significantly greater expression across all metrics relative to the Gq and Gi groups. We regard this trend as consistent with expected construct-specific expression profiles. No significant differences were observed between the viral groups (Gi & Gq) which would be responsive to CNO.

**Supplemental Table 1. EPM + CPP hemizygous animals: Pooled t-tests and Hedges’ g effect sizes (coverage) or Mann-Whitney U tests and Cliff’s delta effect sizes (total cells, dispersion) for DREADD expression metrics across viral groups, with FWER and FDR corrections.**

| **Metric** | **Group1** | **Group2** | **Effect** | **p (raw)** | **p (FWER)** | **p (FDR)** |
| --- | --- | --- | --- | --- | --- | --- |
| Total cells | Gi | Gq | 0.351290685 | 0.015115494 | 0.133338474 | 0.068019724 |
| Total cells | Gi | mC | -0.038961039 | 0.886773624 | 1 | 0.935622689 |
| Total cells | Gq | mC | -0.44973545 | 0.073172881 | 0.512210165 | 0.168154419 |
| Coverage | Gi | Gq | 0.020778233 | 0.935622689 | 1 | 0.935622689 |
| Coverage | Gi | mC | 0.425559987 | 0.30335444 | 1 | 0.546037992 |
| Coverage | Gq | mC | 0.32468581 | 0.438783508 | 1 | 0.658175262 |
| Dispersion | Gi | Gq | 0.369248036 | 0.014815386 | 0.133338474 | 0.068019724 |
| Dispersion | Gi | mC | 0.437229437 | 0.074735297 | 0.512210165 | 0.168154419 |
| Dispersion | Gq | mC | 0.058201058 | 0.835109049 | 1 | 0.935622689 |

**Supplemental Table 2. FST hemizygous animals: Pooled t-tests and Hedges’ g effect sizes (dispersion) or Mann-Whitney U tests and Cliff’s delta effect sizes (total cells, coverage) for DREADD expression metrics across viral groups, with FWER and FDR corrections.**

| **Metric** | **Group1** | **Group2** | **Effect** | **p (raw)** | **p (FWER)** | **p (FDR)** |
| --- | --- | --- | --- | --- | --- | --- |
| Total cells | Gi | Gq | -0.111111111 | 0.710822257 | 1 | 0.83022527 |
| Total cells | Gi | mC | -0.8 | 0.003748772 | 0.015425751 | 0.005623157 |
| Total cells | Gq | mC | -0.828571429 | 0.00308515 | 0.015425751 | 0.00555327 |
| Coverage | Gi | Gq | 0.015873016 | 1 | 1 | 1 |
| Coverage | Gi | mC | -0.955555556 | 0.000520244 | 0.00364171 | 0.001560733 |
| Coverage | Gq | mC | -0.914285714 | 0.000719868 | 0.00431921 | 0.001619704 |
| Dispersion | Gi | Gq | 0.162596601 | 0.737978018 | 1 | 0.83022527 |
| Dispersion | Gi | mC | -2.421316627 | 3.77E-05 | 0.000339504 | 0.000339504 |
| Dispersion | Gq | mC | -2.27484464 | 0.000206588 | 0.001652703 | 0.000929645 |
